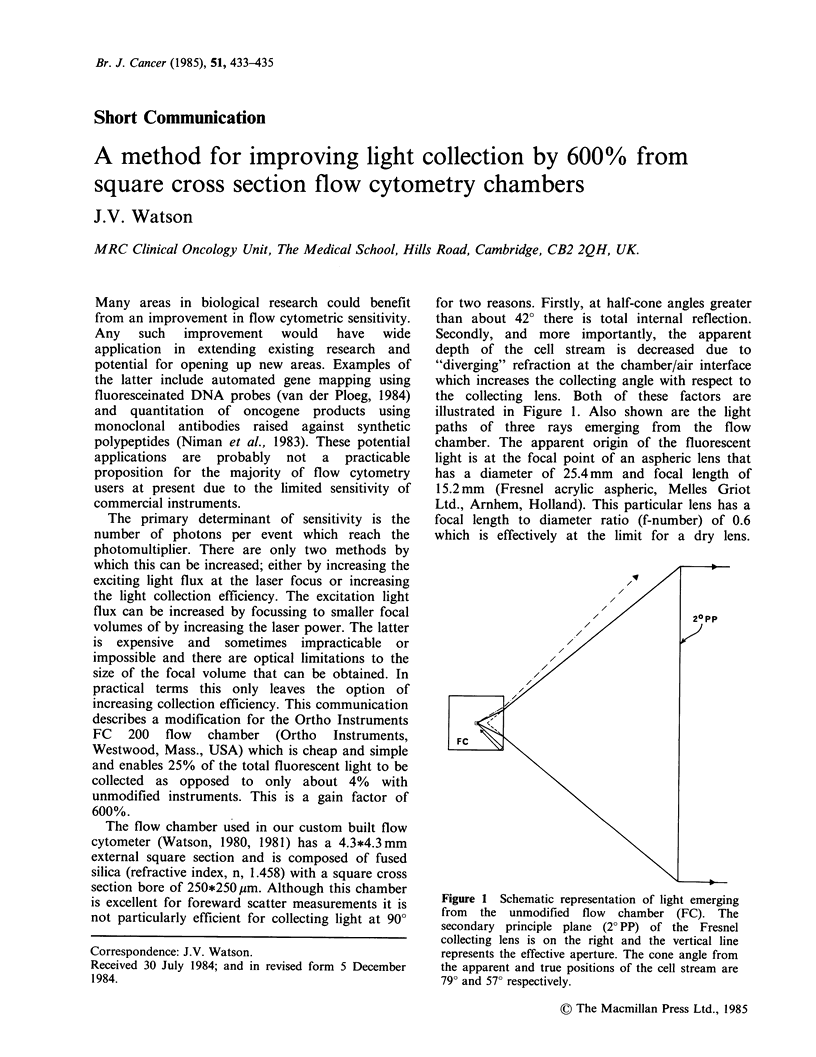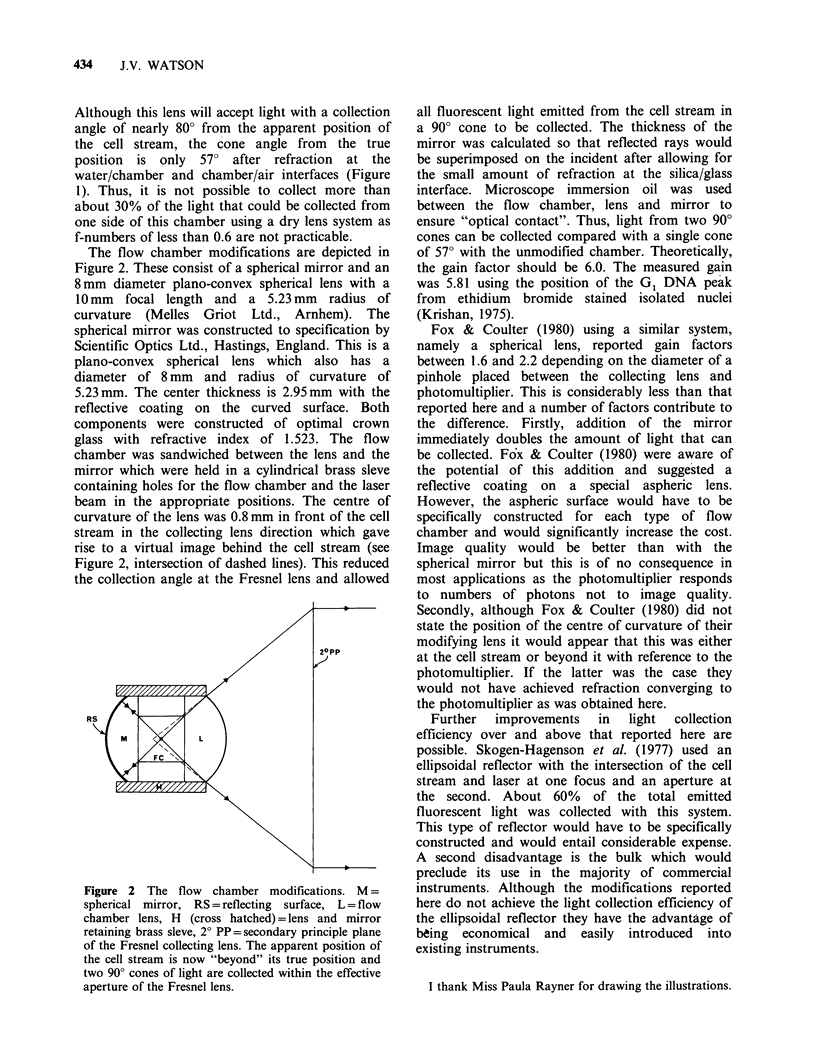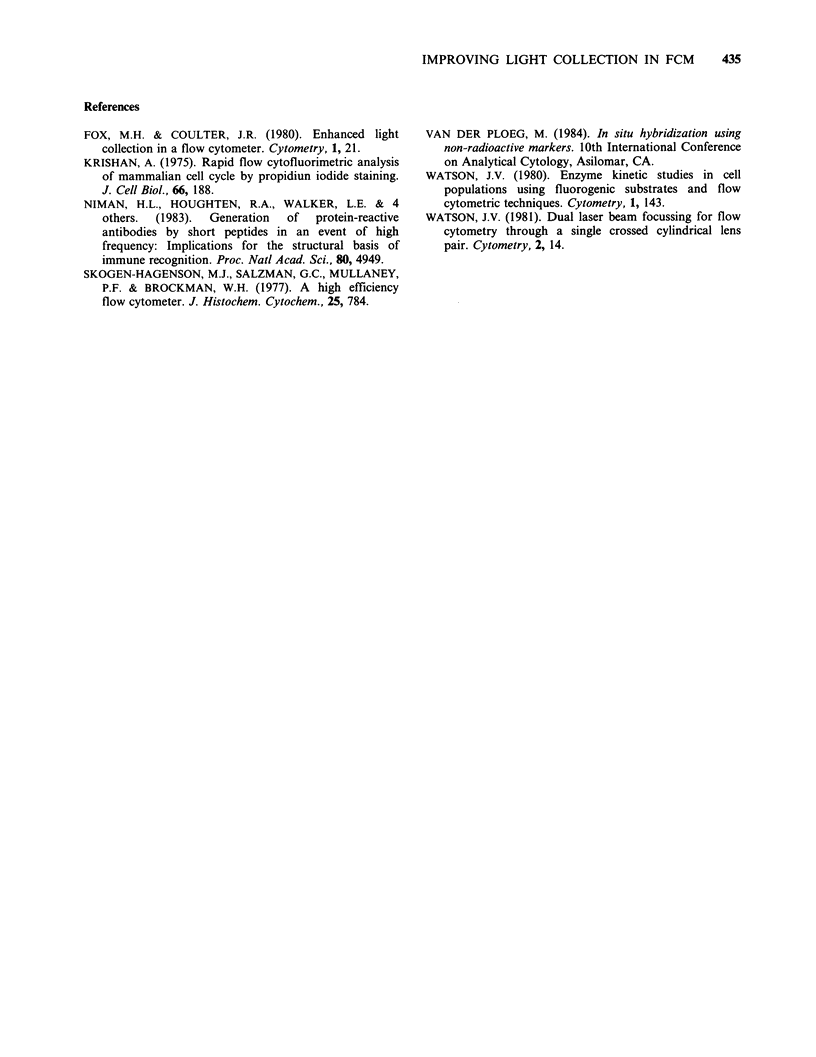# A method for improving light collection by 600% from square cross section flow cytometry chambers.

**DOI:** 10.1038/bjc.1985.60

**Published:** 1985-03

**Authors:** J. V. Watson


Br. J. Cancer (1985), **51**, 433–435

**Short Communication**

### A method for improving light collection by 600% from square cross section flow cytometry chambers

J.V. Watson

*MRC Clinical Oncology Unit, The Medical School, Hills Road, Cambridge, CB2 2QH, UK.*

Many areas in biological research could benefit from an improvement in flow cytometric sensitivity. Any such improvement would have wide application in extending existing research and potential for opening up new areas. Examples of the latter include automated gene mapping using fluoresceinated DNA probes (van der Ploeg, 1984) and quantitation of oncogene products using monoclonal antibodies raised against synthetic polypeptides (Niman *et al.,* 1983). These potential applications are probably not a practicable proposition for the majority of flow cytometry users at present due to the limited sensitivity of commercial instruments.

The primary determinant of sensitivity is the number of photons per event which reach the photomultiplier. There are only two methods by which this can be increased; either by increasing the exciting light flux at the laser focus or increasing the light collection efficiency. The excitation light flux can be increased by focussing to smaller focal volumes of by increasing the laser power. The latter is expensive and sometimes impracticable or impossible and there are optical limitations to the size of the focal volume that can be obtained. In practical terms this only leaves the option of increasing collection efficiency. This communication describes a modification for the Ortho Instruments FC 200 flow chamber (Ortho Instruments, Westwood, Mass., USA) which is cheap and simple and enables 25% of the total fluorescent light to be collected as opposed to only about 4% with unmodified instruments. This is a gain factor of 600%.

The flow chamber used in our custom built flow cytometer (Watson, 1980, 1981) has a 4.3*4.3 mm external square section and is composed of fused silica (refractive index, n, 1.458) with a square cross section bore of 250*250 $\mu$m. Although this chamber is excellent for foreward scatter measurements it is not particularly efficient for collecting light at 90° for two reasons. Firstly, at half-cone angles greater than about 42° there is total internal reflection. Secondly, and more importantly, the apparent depth of the cell stream is decreased due to "diverging" refraction at the chamber/air interface which increases the collecting angle with respect to the collecting lens. Both of these factors are illustrated in Figure 1. Also shown are the light paths of three rays emerging from the flow chamber. The apparent origin of the fluorescent light is at the focal point of an aspheric lens that has a diameter of 25.4 mm and focal length of 15.2 mm (Fresnel acrylic aspheric, Melles Griot Ltd., Arnhem, Holland). This particular lens has a focal length to diameter ratio (f-number) of 0.6 which is effectively at the limit for a dry lens.

**Figure 1** Schematic representation of light emerging from the unmodified flow chamber (FC). The secondary principle plane (2° PP) of the Fresnel collecting lens is on the right and the vertical line represents the effective aperture. The cone angle from the apparent and true positions of the cell stream are 79° and 57° respectively.

Correspondence: J.V. Watson.
Received 30 July 1984; and in revised form 5 December 1984.

© The Macmillan Press Ltd., 1985

Although this lens will accept light with a collection angle of nearly 80° from the apparent position of the cell stream, the cone angle from the true position is only 57° after refraction at the water/chamber and chamber/air interfaces (Figure 1). Thus, it is not possible to collect more than about 30% of the light that could be collected from one side of this chamber using a dry lens system as f-numbers of less than 0.6 are not practicable.

The flow chamber modifications are depicted in Figure 2. These consist of a spherical mirror and an 8 mm diameter plano-convex spherical lens with a 10 mm focal length and a 5.23 mm radius of curvature (Melles Griot Ltd., Arnhem). The spherical mirror was constructed to specification by Scientific Optics Ltd., Hastings, England. This is a plano-convex spherical lens which also has a diameter of 8 mm and radius of curvature of 5.23 mm. The center thickness is 2.95 mm with the reflective coating on the curved surface. Both components were constructed of optimal crown glass with refractive index of 1.523. The flow chamber was sandwiched between the lens and the mirror which were held in a cylindrical brass sleve containing holes for the flow chamber and the laser beam in the appropriate positions. The centre of curvature of the lens was 0.8 mm in front of the cell stream in the collecting lens direction which gave rise to a virtual image behind the cell stream (see Figure 2, intersection of dashed lines). This reduced the collection angle at the Fresnel lens and allowed

**Figure 2** The flow chamber modifications. M = spherical mirror, RS = reflecting surface, L = flow chamber lens, H (cross hatched) = lens and mirror retaining brass sleve, 2° PP = secondary principle plane of the Fresnel collecting lens. The apparent position of the cell stream is now "beyond" its true position and two 90° cones of light are collected within the effective aperture of the Fresnel lens.

all fluorescent light emitted from the cell stream in a 90° cone to be collected. The thickness of the mirror was calculated so that reflected rays would be superimposed on the incident after allowing for the small amount of refraction at the silica/glass interface. Microscope immersion oil was used between the flow chamber, lens and mirror to ensure "optical contact". Thus, light from two 90° cones can be collected compared with a single cone of 57° with the unmodified chamber. Theoretically, the gain factor should be 6.0. The measured gain was 5.81 using the position of the $G_1$ DNA peak from ethidium bromide stained isolated nuclei (Krishan, 1975).

Fox & Coulter (1980) using a similar system, namely a spherical lens, reported gain factors between 1.6 and 2.2 depending on the diameter of a pinhole placed between the collecting lens and photomultiplier. This is considerably less than that reported here and a number of factors contribute to the difference. Firstly, addition of the mirror immediately doubles the amount of light that can be collected. Fox & Coulter (1980) were aware of the potential of this addition and suggested a reflective coating on a special aspheric lens. However, the aspheric surface would have to be specifically constructed for each type of flow chamber and would significantly increase the cost. Image quality would be better than with the spherical mirror but this is of no consequence in most applications as the photomultiplier responds to numbers of photons not to image quality. Secondly, although Fox & Coulter (1980) did not state the position of the centre of curvature of their modifying lens it would appear that this was either at the cell stream or beyond it with reference to the photomultiplier. If the latter was the case they would not have achieved refraction converging to the photomultiplier as was obtained here.

Further improvements in light collection efficiency over and above that reported here are possible. Skogen-Hagenson *et al.* (1977) used an ellipsoidal reflector with the intersection of the cell stream and laser at one focus and an aperture at the second. About 60% of the total emitted fluorescent light was collected with this system. This type of reflector would have to be specifically constructed and would entail considerable expense. A second disadvantage is the bulk which would preclude its use in the majority of commercial instruments. Although the modifications reported here do not achieve the light collection efficiency of the ellipsoidal reflector they have the advantage of being economical and easily introduced into existing instruments.

I thank Miss Paula Rayner for drawing the illustrations.

### References

FOX, M.H. & COULTER, J.R. (1980). Enhanced light collection in a flow cytometer. *Cytometry*, **1**, 21.

KRISHAN, A. (1975). Rapid flow cytofluorimetric analysis of mammalian cell cycle by propidiun iodide staining. *J. Cell Biol.*, **66**, 188.

NIMAN, H.L., HOUGHTEN, R.A., WALKER, L.E. & 4 others. (1983). Generation of protein-reactive antibodies by short peptides in an event of high frequency: Implications for the structural basis of immune recognition. *Proc. Natl Acad. Sci.*, **80**, 4949.

SKOGEN-HAGENSON, M.J., SALZMAN, G.C., MULLANEY, P.F. & BROCKMAN, W.H. (1977). A high efficiency flow cytometer. *J. Histochem. Cytochem.*, **25**, 784.

VAN DER PLOEG, M. (1984). *In situ hybridization using non-radioactive markers.* 10th International Conference on Analytical Cytology, Asilomar, CA.

WATSON, J.V. (1980). Enzyme kinetic studies in cell populations using fluorogenic substrates and flow cytometric techniques. *Cytometry*, **1**, 143.

WATSON, J.V. (1981). Dual laser beam focussing for flow cytometry through a single crossed cylindrical lens pair. *Cytometry*, **2**, 14.